# Radiation Dose and Fluoroscopy Time of Extracranial Carotid Artery Stenting: Elective vs. Emergency Treatment Including Combined Mechanical Thrombectomy in Tandem Occlusion

**DOI:** 10.1007/s00062-023-01288-w

**Published:** 2023-06-01

**Authors:** Robert Forbrig, Yigit Ozpeynirci, Thomas David Fischer, Christoph G. Trumm, Thomas Liebig, Robert Stahl

**Affiliations:** Institute of Neuroradiology, University Hospital, LMU Munich, Marchioninistr. 15, 81377 Munich, Germany

**Keywords:** Angioplasty, Carotid stenosis, Endovascular procedures, Radiation exposure, Stroke

## Abstract

**Purpose:**

Fluoroscopically guided endovascular carotid artery stenting (CAS) of extracranial carotid stenosis (ECS) is a reasonable alternative to carotid endarterectomy in selected patients. Diagnostic reference levels (DRL) for this common neurointervention have not yet been defined and respective literature data are sparse. We provide detailed dosimetrics for useful expansion of the DRL catalogue.

**Methods:**

A retrospective single-center study of patients undergoing CAS between 2013 and 2021. We analyzed dose area product (DAP) and fluoroscopy time considering the following parameters: indications for CAS, semielective/elective versus emergency including additional mechanical thrombectomy (MT) in extracranial/intracranial tandem occlusion, etiology of ECS (atherosclerotic vs. radiation-induced), periprocedural features, e.g., number of applied stents, percutaneous transluminal angioplasty (PTA) and MT maneuvers, and dose protocol. Local DRL was defined as 75% percentile of the DAP distribution.

**Results:**

A total of 102 patients were included (semielective/elective CAS *n* = 75, emergency CAS *n* = 8, CAS + MT *n* = 19). Total median DAP was 78.2 Gy cm^2^ (DRL 117 Gy cm^2^). Lowest and highest median dosimetry values were documented for semielective/elective CAS and CAS + MT (DAP 49.1 vs. 146.8 Gy cm^2^, fluoroscopy time 27.1 vs. 43.8 min; *p* < 0.005), respectively. Dosimetrics were significantly lower in patients undergoing 0–1 PTA maneuvers compared to ≥ 2 maneuvers (*p* < 0.05). Etiology of ECS, number of stents and MT maneuvers had no significant impact on dosimetry values (*p* > 0.05). A low-dose protocol yielded a 33% reduction of DAP.

**Conclusion:**

This CAS study suggests novel local DRLs for both elective and emergency cases with or without intracranial MT. A dedicated low-dose protocol was suitable for substantial reduction of radiation dose.

## Introduction

Extracranial carotid stenosis (ECS) is usually located at the proximal segment of the internal carotid artery and/or distal portion of the common carotid artery. Beside carotid endarterectomy (CEA), fluoroscopically guided endovascular carotid artery stenting (CAS) is an effective and safe treatment modality [1]. Based on ECS guidelines [2] the indications for treatment depend on i) patient age, ii) etiology (e.g., atherosclerotic, radiation-induced), iii) degree of stenosis, and iv) clinical symptoms. A distinction can be made between purely elective (e.g., progressive asymptomatic ECS) or semielective (symptomatic ECS, e.g., amaurosis fugax or transient ischemic attack in the downstream brain-supplying territory) and emergency treatment indications (e.g., acute carotid occlusion or combined extracranial/intracranial tandem occlusion). Recent data show that in patients with symptomatic ECS the risk of stroke recurrence ≥ 30 days after treatment or at 2‑year follow-up is equally low for both treatment modalities [3, 4]. In contrast, the periprocedural/postprocedural (days 0–30) stroke and mortality risks increase significantly in patients > 70 years treated by CAS compared with CEA [4]; however, the absolute risk of complications is equally low for CAS and CEA, thus qualifying both procedures if carefully indicated [1]. Furthermore, in patients with acute tandem occlusion, CAS is often primarily required as access route for intracranial mechanical thrombectomy (MT).

The national guidelines for radiation protection were revised in 2019 and require full documentation of and compliance with diagnostic reference levels (DRL) for X‑ray procedures [5]. In this context, the dose descriptor dose area product (DAP) is usually applied for fluoroscopically guided endovascular methods. The Federal Office for Radiation Protection has updated the DRL catalogue for fluoroscopically guided interventions in 2022 [6]. Regarding neuroangiography, this catalogue includes only DRLs for endovascular stroke and aneurysm treatment. Other common neurointerventions such as CAS are not yet included. In addition to the documentation of DAP, fluoroscopy time also plays a major role in fluoroscopically guided endovascular procedures as the risk of periprocedural complications is directly associated with the intervention duration [7].

In recent years, several dosimetry studies have been published to usefully extend the DRL catalogue for specific neuroangiographic indications. These include DRLs for intracranial MT in acute stroke patients as well as embolization of intracranial aneurysms and arteriovenous malformations [8–17]. Regarding CAS, however, dosimetry data are sparse and particularly do not contain patients with acute tandem occlusion [18–22].

The aim of this retrospective single-center study was to evaluate radiation dose and fluoroscopy time in patients with ECS undergoing CAS, considering different indications (elective vs. emergency CAS including additional MT in acute tandem occlusion), periprocedural parameters, and dose protocol settings. Our locally recorded DRLs may be useful to introduce novel national DRLs in the field of endovascular carotid revascularization.

## Material and Methods

### Patients

To identify qualifying subjects, we conducted a database search for the specific procedure key “stent angioplasty of an extracranial brain supplying artery” using the radiology information system of our institution. Then, both the etiology of ECS and indication for CAS were documented by using a full text search of the corresponding written reports as well as the respective operations and procedure codes.

**Fig. 1:**
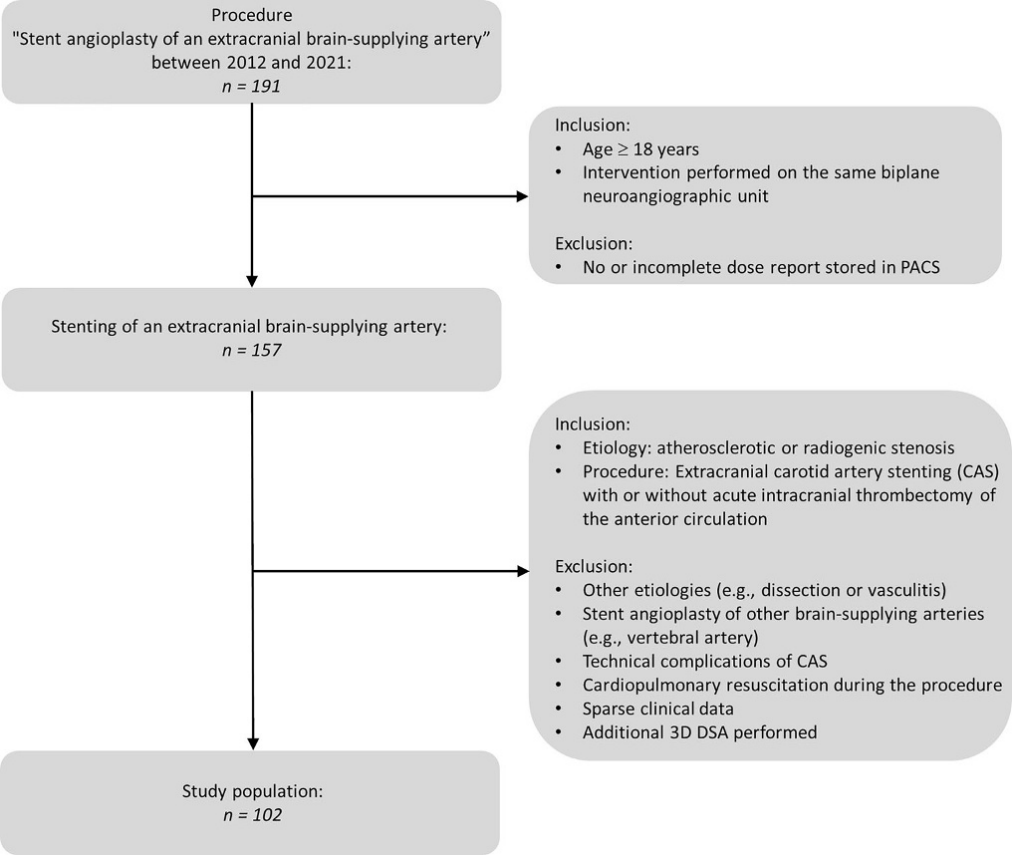
Flow chart of inclusion and exclusion criteria. *CAS* carotid artery stenting, *DSA* digital subtraction angiography, *ECS* extracranial carotid stenosis, *MT* mechanical thrombectomy, *n* number, *PACS* picture archiving and communication system

We included patients who had undergone CAS due to an atherosclerotic or radiation-induced ECS ≥ 50% with or without additional intracranial MT (in cases of acute tandem occlusion) at our institution between January 2013 and June 2021. Interventions in which technical complications (e.g., displacement of the stent applicator, iatrogenic carotid dissection, etc.) occurred were excluded. Details of the selection process are shown in Fig. 1.

Patients without intracranial MT (i.e., CAS only) were dichotomized in semielective/elective and emergency groups. We also documented the following clinical parameters: age, sex, cardiovascular risk factors and whether CAS was a first or repeated carotid intervention.

### Endovascular Procedure

Endovascular procedures were performed by 5 consultant neuroradiologists with 6 to more than 20 years of experience in interventional neuroradiology. Before the intervention, patients were administered mono (in cases of emergency) or dual antiplatelet therapy. The utilized angiographic system was a biplane angiographic unit (Axiom Artis dBA, Siemens Healthineers, Forchheim, Germany). A transfemoral approach was used in each patient. For vessel visualization, a non-ionic iodinated contrast agent was applied (iomeprol 300 mg iodine/ml; Imeron, Bracco Imaging, Konstanz, Germany). The angiographic workflow routinely comprised initial and final DSA acquisitions of both the extracranial carotid lesion and downstream brain-supplying arteries on standard anterior-posterior and lateral projections with a field of view (FOV) of 22–32 cm and pulsed fluoroscopy of 7.5 f/s.

The use of an embolic protection device as well as the choice of the respective stent and MT device (in acute tandem occlusion) were at the neurointerventionist’s discretion. In general, the common carotid artery was catheterized using an 8‑French guiding catheter (e.g., Vista Brite Tip, Cordis, Santa Clara, CA, USA). Then, the optimal angle for ECS visualization was sought in targeted projections with a FOV of 11–22 cm, and a suitable self-expanding stent (e.g., Carotid Wallstent, Boston Scientific, Marlborough, MA, USA) was carefully advanced through the stenosis and deployed. If the stent did not appropriately cover the stenosis, placement of another stent was necessary. In the case of insufficient stent expansion, one or several postdilatation maneuver(s) were performed by inflation of a suitable percutaneous transluminal angioplasty (PTA) balloon (e.g., Emerge, Boston Scientific).

The technique of intracranial MT was reported elsewhere [23]. In brief, in addition to the extracranially placed guiding catheter, a combination of an aspiration catheter (e.g., 5 or 6 F SOFIA, MicroVention Terumo, Aliso Viejo, CA, USA) and microcatheter (e.g., Rebar 18, Medtronic, Dublin, Ireland) was placed intracranially next to the occlusion site under fluoroscopy using a 0.014-inch guidewire (e.g., Synchro, Stryker, Kalamazoo, MI, USA). Then, both the guidewire and microcatheter were advanced through the occlusion using a working FOV of 11–22 cm. After removal of the guidewire, the physician deployed a fitting stent retriever (e.g., Solitaire Platinum, Medtronic) at the occlusion site and finally removed the device under continuous aspiration. This maneuver was repeated several times, if necessary, until the vessel was successfully revascularized.

The digital subtraction angiography (DSA) acquisition type comprised two protocols preset by the manufacturer as previously described [12]:

- Low dose (LD): 2 or 4 f/s (arterial phase), 1 f/s (venous phase), kV 73, pulse width 50 ms, dose 1820 μGy/*p*.
- Normal dose (ND): 2 or 4 f/s (arterial phase), 1 f/s (venous phase), kV 73, pulse width 100 ms, dose 3000 μGy/*p*.

The two dose protocols were used at the neurointerventionist’s discretion. If both protocols were used during an intervention, we documented mixed dose (MD = LD and ND) for reasons of simplification.

Regarding CAS, we documented the number of applied stents and dichotomized the required balloon PTA maneuvers into groups 0–1 and ≥ 2. Technical success was defined as < 30% residual stenosis. In acute tandem occlusion, the intracranial MT maneuver count was dichotomized into groups 1–2 and ≥ 3. Technical success was defined as modified thrombolysis in cerebral ischemia (mTICI) score 2b or 3 [24].

### Radiation Metrics

All imaging data and dose reports were retrieved from the dedicated picture archiving and communication system (Visage Imaging 7.1, Visage Imagin, Berlin, Germany) and reviewed by two experienced neuroradiologists with 5 (R.S.) and 12 (R.F.) years of experience in diagnostic and interventional neuroradiology.

The following parameters were evaluated: DSA acquisition count, DSA protocol, fluoroscopy time and DAP (representing a surrogate measure of energy delivered to patients [25]), and DSA DAP. The individual total DAP was calculated by summing fluoroscopy and DSA DAP. Data of DSA acquisition count, fluoroscopy time, and DAP were documented by summing respective values of both X‑ray tubes (biplane mode).

Furthermore, the impact of different DSA protocols on DAP was investigated. In detail, the total DAP was compared between LD, ND, and MD (both LD and ND DSA acquisitions) groups, and the individual dose index was calculated using the following formula [12]:

$$Dose\,\textit{index}=DSADAP/DSA\,\textit{acquisition}\,\textit{count}$$

### Statistics

Continuous data are provided as mean ± standard deviation (95% confidence interval) and/or median (25%; 75% interquartile range). Distribution of total individual DAP, fluoroscopy time, and individual mean dose index were initially assessed for normality applying the Shapiro-Wilk test considering indications for CAS, etiology of ECS, number of PTA and MT maneuvers, DSA acquisition count, and DSA protocol. The local DRL was defined as 75% percentile of the DAP distribution [26]. Although DAP and fluoroscopy time values were non-normally distributed, we also calculated the respective mean values to ensure comparability with other studies. Categorical data are reported as counts and percent. Intergroup comparison of three groups was performed with univariate analysis of variance (ANOVA) and Kruskal-Wallis tests. If statistically significant differences occurred, single posttest comparisons were performed using the t‑test and Mann-Whitney‑U test with Bonferroni’s correction for multiple comparisons. Comparison of two groups was performed with t‑tests or Wilcoxon-Mann-Whitney tests.

The Spearman rank correlation analysis was applied to investigate the impact of MT maneuvers on DAP and fluoroscopy time.

Differences in the frequency of cardiovascular risk factors between study groups were assessed with χ^2^-tests. In cases of significance, post hoc pairwise Fisher tests with Bonferroni correction were conducted.

Analysis was performed using R (R Core Team. URL https://www.R-project.org/). A level of significance of α = 0.05 was used throughout the study.

## Results

### Patient Characteristics

Patient characteristics are summarized in Table 1. We identified 102 patients with ECS undergoing CAS at our institution between 2013 and 2021 according to the inclusion and exclusion criteria. Of the patients 83/102 (81.4%) received CAS alone, with an emergency treatment indication in 8/83 (9.6%) patients. Of the remainder, 41/75 (54.7%) patients required urgent treatment within a few days (i.e., semielective), 34/75 (45.3%) patients were purely elective cases and 19/102 (18.6%) patients presented with acute extracranial/intracranial tandem occlusion, consequently undergoing both CAS and MT.

**Table 1:** Patient characteristics

Age, mean (range)	69.3 ± 10.4 years (33–93)		
*Sex*	42 females (41.2%), 60 males (58.8%)		
*Indication*	Semielective/Elective CAS (*n* = 75)	Emergency CAS (*n* = 8)	CAS and MT due to acute tandem occlusion (*n* = 19)
*Etiology of ECS* ^a^			
Atherosclerotic	52 (69.3%)	8 (100.0%)	19 (100.0%)
Radiation-induced	23 (30.7%)	0 (0.0%)	0 (0.0%)
*Cardiovascular risk factors* ^a^			
Diabetes	19 (25.3%)	4 (50.0%)	4 (21.1%)
Hypercholesterolemia	43 (57.3%)	1 (12.5%)	2 (10.5%)
Hypertension	59 (78.7%)	6 (75.0%)	13 (68.4%)
Nicotine abuse	43 (57.3%)	3 (37.5%)	4 (21.1%)
*First treatment* ^a^	45 (60.0%)	8 (100.0%)	18 (94.7%)
*Repeated treatment* ^a^			
After prior CEA	19 (25.3%)	0 (0.0%)	0 (0.0%)
After prior CAS	11 (14.7%)	0 (0.0%)	1 (5.3%)

*CAS* carotid artery stenting, *CEA* carotid endarterectomy, *ECS* extracranial carotid stenosis, *MT* mechanical thrombectomy

^a^ Percentages refer to the total number of cases in the corresponding indication subgroup

In the CAS-only group, ECS was caused by severe atherosclerosis in 60/83 (77.3%) and radiation-induced vessel wall thickening in 23/83 (27.7%) patients. The distribution of underlying tumor entities in patients with radiogenic ECS was as follows: carcinoma of the floor of the mouth and tongue base (*n* = 8); tonsillar carcinoma (*n* = 6); papillary thyroid carcinoma (*n* = 3); pharyngeal carcinoma (*n* = 2); cancer of unknown primary (*n* = 2); and one case each of non-Hodgkin’s lymphoma and laryngeal carcinoma. The respective CAS procedure was conducted after a median of 155 months after completion of radiotherapy. Each patient with radiation-induced ECS presented with a semielective/elective indication for CAS. All patients requiring CAS due to an emergency indication (*n* = 27; without or without additional MT) showed an underlying atherosclerotic ECS.

Both hypercholesterolemia and nicotine abuse were significantly more frequent (*p* < 0.05) in patients undergoing semielective/elective CAS (each 43/75, 57.3%) compared to the other groups.

CAS was the first treatment in 8/8 (100%) patients presenting with an emergency indication and in 18/19 (94.7%) patients with acute tandem occlusion. In contrast, 30/75 (40%) patients with a semielective/elective indication presented with a history of at least 1 prior ECS treatment.

### Endovascular Procedure and Technical Success

In the CAS-only group 1 stent was applied in 71/83 (85.5%) patients, with 0–1 PTA maneuvers in 45/71 (63.4%) and ≥ 2 PTA maneuvers in 26/71 (36.6%) patients. 11/83 (13.3%) patients received 2 stents, with 0–1 PTA maneuvers in 4/11 (36.4%) and ≥ 2 PTA maneuvers in 7/11 (63.6%) patients. 1/83 patients (1.2%) received 4 stents without need of additional PTA maneuvers.

The high number of cases with more than one implanted carotid stent (14.5%, 12/83 cases) was due to the fact that the majority of these patients showed a long segment postradiogenic stenosis (83.3%, 10/12 cases). Consequently, 10/23 (43.5%) postradiogenic stenoses were treated with more than 1 stent.

Technical success of CAS (residual stenosis < 30%) was 94.7% (*n* = 71/75) in the semielective/elective group and 87.5% (*n* = 7/8) in the emergency group. A residual stenosis of 30–49% was documented in 4/75 (5.3%) and 1/8 (12.5%) patients, respectively.

Regarding acute tandem occlusion, additionally to extracranial CAS, 1–2 intracranial MT maneuvers were performed in 15/19 (78.9%) patients and ≥ 3 MT maneuvers in 4/19 (21.1%) patients. Technical success of MT was 100%. In detail, mTICI scores 2b and 3 were achieved in 15/19 (78.9%) and 4/19 (21.1%) patients, respectively.

### Radiation Dose and Fluoroscopy Time

**Fig. 2:**
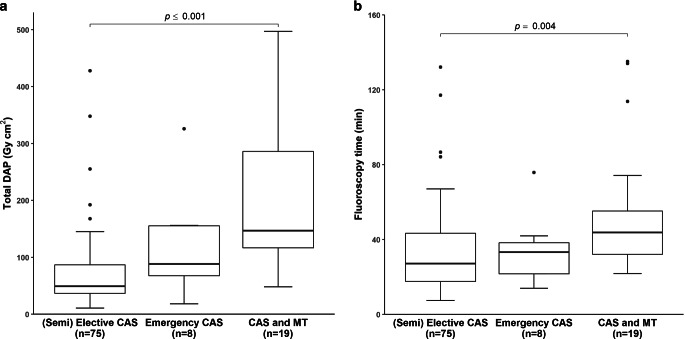
Radiation dose (**a**) and fluoroscopy time (**b**) with respect to different indications for CAS. Values are shown as median (25%; 75% percentile). Median dosimetry values were significantly higher in patients undergoing CAS and MT due to acute extra-/intracranial tandem occlusion compared to semielective/elective CAS-only procedures. *CAS* carotid artery stenting, *DAP* dose area product, *MT* mechanical thrombectomy, *n* number

**Fig. 3:**
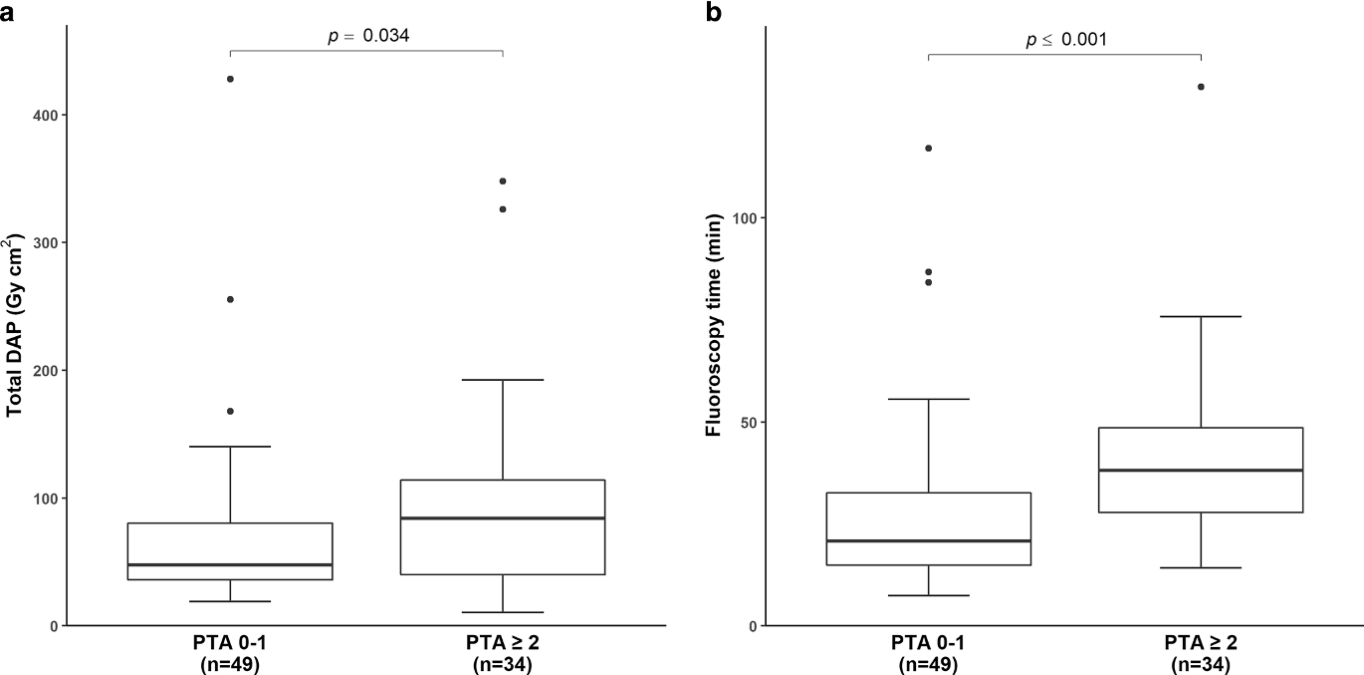
Radiation dose (**a**) and fluoroscopy time (**b**) of CAS-only interventions with respect to the number of PTA maneuvers. Values are shown as median (25%; 75% percentile). Median dosimetry values were significantly higher in patients with ≥ 2 PTA maneuvers compared to those with 0–1 maneuvers. *DAP* dose area product, *n* number, *PTA* percutaneous transluminal angioplasty

**Fig. 4:**
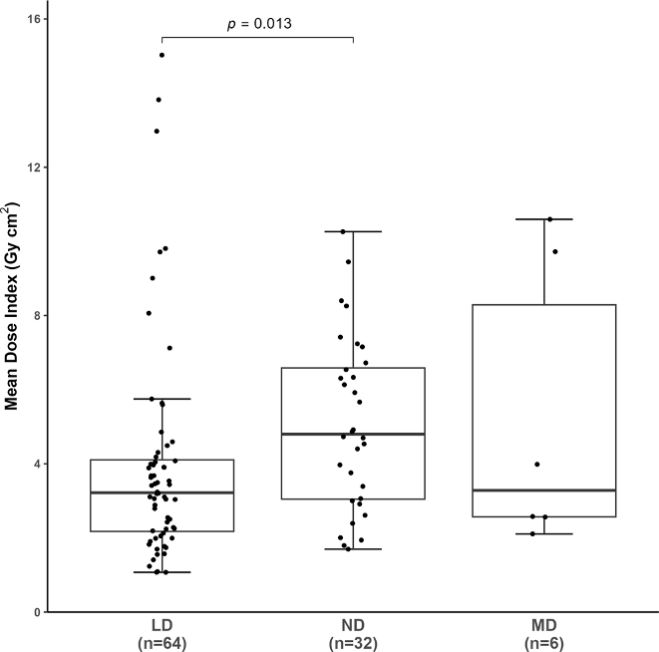
Dose index with respect to different DSA protocols. Values are shown as median (25%; 75% percentile). The median dose index was significantly lower in the LD group when compared to the ND group. *LD* low dose, *ND* normal dose, *MD* mixed dose, *n* number

Major dosimetry results are shown in Table 2 and Figs. 2, 3, and 4. Total mean and median DAP were 103.2 ± 98.2 Gy cm^2^ and 78.2 (25%; 75% interquartile range: 40.0; 117.0) Gy cm^2^, respectively. Both median DAP and fluoroscopy time were significantly higher in patients undergoing CAS + MT as in CAS-only patients with a semielective/elective indication (DAP 146.8 versus 49.1 Gy cm^2^, fluoroscopy time 43.8 versus 27.1 min; *p* < 0.005, each).

**Table 2:** DAP and fluoroscopy time regarding different subgroups and DSA protocols

Total DAP (*n* = 102) (Gy cm^2^)	103.2 ± 98.2 (83.9–122.5) (mean)	78.2 (40.0; 117.0) (median)		
*Indication*	Semielective/Elective CAS (*n* = 75)	Emergency CAS (*n* = 8)	CAS and MT due to acute tandem occlusion (*n* = 19)	*P*-Value
Mean DAP ^a^ (Gy cm^2^)	75.3 ± 67.7 (59.7–90.9)	118.2 ± 97.2 (36.9–199.4)	206.9 ± 130.2 (144.2–269.6)	**Anova**: ***p*** ***<*** ***0.001*** ^**c**^ (Semi) Elective vs. Emergency CAS: *p* *=* *0.529* (Semi) Elective CAS vs. CAS + MT: ***p*** ***<*** ***0.001*** Emergency CAS vs. CAS + MT: ***p*** ***=*** ***0.044***
Median DAP ^b^ (Gy cm^2^)	49.1 (36.7; 86.7)	88.3 (67.4; 155.2)	146.8 (116.8; 286.1)	**Kruskal-Wallis-Test**: ***p*** ***<*** ***0.001*** ^**d**^ (Semi) Elective vs. Emergency CAS: *p* *=* *0.460* (Semi) Elective CAS vs CAS + MT: ***p*** ***<*** ***0.001*** Emergency CAS vs. CAS + MT: *p* *=* *0.250*
Mean FL time ^a^ (min)	32.9 ± 22.9 (27.7–38.2)	34.7 ± 19.1 (18.7–50.7)	54.5 ± 35.2 (37.5–71.5)	**Anova**: ***p*** ***=*** ***0.005*** ^**c**^ (Semi) Elective vs. Emergency CAS: *p* *=* *1.00* (Semi) Elective CAS vs. CAS + MT: ***p*** ***=*** ***0.004*** Emergency CAS vs. CAS + MT: *p* *=* *0.199*
Median FL time ^b^ (min)	27.1 (17.6; 43.3)	33.2 (21.6; 38.2)	43.8 (32.1; 55.2)	**Kruskal-Wallis-Test**: ***p*** ***=*** ***0.005*** ^**d**^ (Semi) Elective vs. emergency CAS: *p* *=* *1.00* (Semi) Elective CAS vs. CAS + MT: ***p*** ***=*** ***0.004*** Emergency CAS vs. CAS + MT: *p* *=* *0.225*
*PTA manoeuvres*	0–1 (*n* = 49)	≥ 2 (*n* = 34)	–	–
Mean DAP ^a^ (Gy cm^2^)	68.8 ± 66.9 (49.6–88.0)	94.7 ± 75.9 (68.2–121.2)	–	*p* *=* *0.114* ^e^
Median DAP ^b^ (Gy cm^2^)	47.5 (36.1; 80.4)	84.1 (40.0; 114.2)	–	***p*** ***=*** ***0.034*** ^f^
Mean FL time ^a^ (min)	27.5 ± 21.4 (21.4–33.6)	41.1 ± 21.4 (33.5–48.8)	–	***p*** ***=*** ***0.006*** ^e^
Median FL time ^b^ (min)	20.8 (14.9; 32.6)	38.1 (27.8; 48.6)	–	***p*** ***<*** ***0.0001*** ^f^
*DSA protocol*	LD (*n* = 64)	ND (*n* = 32)	MD (*n* = 6)	–
*Median dose index* ^b^ *(Gy cm*^*2*^*)*	3.2 (2.2; 4.1)	4.8 (3.0; 6.6)	3.3 (2.6; 8.3)	**Kruskal-Wallis: *p*** ***=*** ***0.018*** ^**d**^ LD vs. ND: ***p*** ***=*** ***0.013*** LD vs. MD: *p* *=* *1.000* ND vs. MD:* p* *=* *1.000*

*Anova* analysis of variance, *CAS* carotid artery stenting, *DAP* dose area product, *DSA* digital subtraction angiography, *ECS* extracranial carotid stenosis, *FL* fluoroscopy, *LD* low dose, *MD* mixed dose, *min* minutes, *MT* mechanical thrombectomy, *n* number, *ND* normal dose, *PTA* percutaneous transluminal angioplasty

Values of radiation dose and FL time are summed for both X‑ray tubes (biplane mode)

^a^ Mean values are shown as mean ± standard deviation (95% confidence interval)

^b^ Median values are shown as median (25%; 75% percentile). Significant *p* values in bold. Post hoc pairwise intergroup comparisons with Bonferroni correction were performed with

^c^ t-tests and

^d^ Wilcoxon-Mann-Whitney tests

^e^ t-test

^f^ Wilcoxon-Mann-Whitney test

With respect to the CAS-only group, we documented significantly higher median dosimetry values in patients undergoing ≥ 2 PTA maneuvers when compared to patients in whom 0–1 PTA maneuvers were carried out (*p* < 0.05, each; Table 2). Median DAP was higher in patients with atherosclerotic ECS than in patients with radiation-induced ECS (70.9 versus 49.1 Gy cm^2^), whereas median fluoroscopy time tended to be lower in the former group (25.9 vs. 33.6 min, *p* > 0.05, each). Neither the number of applied stents nor prior ECS treatment had a significant impact on dosimetrics (*p* > 0.05, each).

Regarding acute tandem occlusion, we observed a trend towards higher dosimetry values in patients undergoing ≥ 3 MT maneuvers (*p* > 0.05, each).

A LD DSA protocol was applied in 64/102 (62.7%), a ND protocol in 32/102 (31.4%), and a MD protocol in 6/102 (5.9%) patients. The median DSA acquisition count did not significantly differ between groups (LD 17.0, ND 14.0, MD 15.0) (*p* > 0.05). Regarding the distribution of radiation dose, total median DAP was 78.2 (39.5; 117.0) Gy cm^2^ for the LD group and 81.1 (45.0; 116.0) Gy cm^2^ for the ND group. These values were statistically not significantly different (*p* > 0.05). The median dose index was significantly lower in the LD group compared to the ND group (3.2 versus 4.8 Gy cm^2^; *p* = 0.013).

## Discussion

In this retrospective single center study we report detailed dosimetrics for the endovascular treatment of CAS due to ECS in 102 patients between 2013 and 2021. We believe that our data may be unique for definition of novel DRLs in accordance with the Euratom Basic Safety Standards directive [27]. In particular, we suggest respective local DRLs for typical indications (semielective/elective vs. emergency CAS with or without intracranial MT in cases of acute tandem occlusion), also taking into account several periprocedural and dose protocol settings.

For national DRL definition of fluoroscopically guided endovascular procedures, the ICRP 135 publication demands documentation of radiation dose metrics such as DAP [26]. This dose descriptor is commonly applied in (neuro)interventional dosimetry studies [8, 9, 11–13, 16, 18–20]. In the present study, we observed a total mean and median DAP of 103.2 ± 98.2 Gy cm^2^ and 78.2 (25%; 75% percentile = local DRL: 40.0; 117.0) Gy cm^2^, respectively. These values are in the broader range of other CAS studies (e.g., Shimizo et al. 73–138 Gy cm^2^, Stanišić et al. 54 Gy cm^2^, Majewska et al. 53 Gy cm^2^) [18–20]; however, in these studies authors did not include combined CAS + MT procedures. In this study, we documented significantly higher dosimetry values in the CAS + MT group (median DAP 146.8 Gy cm^2^, local DRL 286.1 Gy cm^2^; fluoroscopy time 43.8 min) as in semielective/elective CAS-only patients (median DAP 49.1 Gy cm^2^, local DRL 86.7 Gy cm^2^; fluoroscopy time 27.1 min). Consequently, when considering the latter group, radiation dose was even lower compared to values reported in the literature while the fluoroscopy time was comparable to published data (e.g., Swerdlow et al. 21–24 min, D’Ercole et al. 28 min) [21, 22].

A comparably higher radiation exposure of combined CAS + MT in acute tandem occlusion is reasonable. Intracranial MT of acute large vessel occlusion itself represents a sometimes technically challenging neurointervention, thus yielding a certain amount of DAP with values ranging between 80–140 Gy cm^2^ according to the literature [8, 11, 13]. Dosimetry values may increase if multiple MT maneuvers are required. In this study, we indeed encountered slightly higher dosimetry values in patients undergoing ≥ 3 MT maneuvers when compared to 1–2 MT maneuvers, even though not reaching statistical significance. Nevertheless, the median DAP boost of 50–100 Gy cm^2^ in the CAS + MT group (when compared to CAS-only procedures) was even slightly lower when compared to MT dosimetry data of other study groups as shown above.

In CAS-only procedures, we observed a trend towards higher median dosimetrics in emergency cases (DAP 88.3 Gy cm^2^, fluoroscopy time 33.2 min) compared to the semielective/elective group. This finding may be attributed to the relatively low sample size of the former group (*n* = 8). Another explanation, however, might be the commonly demanding periprocedural setting in acute stroke patients under conscious sedation (this study: emergency CAS 6/8 patients), possibly yielding substantial motion artifacts and reduced image quality which in turn may necessitate both a longer fluoroscopy time and increased amount of DSA acquisitions. Regarding additional balloon angioplasty, dosimetry values were significantly higher in patients undergoing ≥ 2 PTA maneuvers compared to 0–1 PTA maneuvers (DAP 84.1 versus 47.5 Gy cm^2^, fluoroscopy time 38.1 versus 20.8 min). As the majority of patients presented with underlying atherosclerotic ECS, these findings clearly reflect the different complexity of individual cases with a heterogeneous severity of the commonly calcified carotid plaques. In this context, we also recorded slightly increased dosimetry values in patients receiving more than one carotid stent; however, these differences did not reach statistical significance.

Interestingly, the median fluoroscopy time of postradiogenic ECS cases (*n* = 23) was slightly higher when compared to the atherosclerotic group (*n* = 60, 33.6 versus 25.9 min; *p* > 0.05) although the radiation dose was lower in the former group as expected (49.1 versus 70.9 Gy cm^2^; *p* > 0.05). These findings can possibly be explained by two facts. First, patients with postradiogenic ECS commonly received more than one stent due to a long-distance stenosis (10/23 patients), yielding substantially longer fluoroscopy times during repeated catheterization and stent deployment. Second, because the DAP of pulsed fluoroscopy is relatively low as compared to a DSA run, a longer fluoroscopy time does not necessarily imply a significantly increased radiation dose. Regarding radiation dose optimization, several techniques have been described [12, 13, 16, 18, 21]. For example, Shimizo et al. showed that a reduced fluoroscopy frame rate (4 instead of 7.5 f/s) may yield a substantial reduction of DAP in patients undergoing CAS [18]. To note, the radiation dose in their group with a reduced frame rate was comparable to values obtained in our semielective/elective CAS group using a frame rate of 7.5 f/s. Furthermore, the positive impact of a dedicated LD protocol on radiation dose has recently been reported [12]. Similarly, in the present study this LD protocol yielded a 33% reduction of DAP per DSA acquisition compared to procedures in whom a ND protocol was applied (3.2 versus 4.8 Gy cm^2^). We therefore strongly recommend usage of specific LD protocols, except in cases of devices and/or implants with a low radiopacity. In this situation, a ND protocol should be preferred to improve their visualization (e.g., intra/extra-aneurysmal flow diversion) [12]. Regarding CAS, another technique may be a 3D image fusion of preprocedurally acquired computed tomography and/or magnetic resonance angiography of the aortic arch and supra-aortic arteries with conventional angiography, potentially reducing periprocedural dosimetry values according to Swerdlow et al. [21]; however, in cases of computed tomography, the sum of radiation dose gathered by both modalities may even exceed radiation exposure of the CAS procedure alone. Finally, in general, the angiography system should ideally be equipped with modern soft-/hardware techniques (e.g., ClarityIQ, Philips, Amsterdam, Netherlands [28] or OPTIQ, Siemens Healthineers [29]). Söderman et al. showed that a dedicated noise reduction algorithm may yield a 60% reduction of radiation exposure during neuroangiographic procedures while maintaining image quality [16]. In another study, Guenego et al. reported a 35% reduction of median DAP in acute stroke patients undergoing intracranial MT after installation of a specific dose reduction system [13]. Apart from the abovementioned technical aspects, further important features of radiation dose optimization are i) a high experience of the treating interventionist, ii) a good compliance of the patient (under sufficient sedation or general anesthesia, if needed), and iii) a broad availability of various catheters and devices (e.g., in case of technically challenging vessel anatomy).

Due to the retrospective mono-centric study design, our results have to be interpreted with caution. First, neurointerventions were carried out using only one angiography system from a single manufacturer (Siemens Healthineers), thus generalizability of our findings is limited. Second, our study population is relatively small (*n* = 102) and CAS procedures (with and without additional MT) were carried out in a rather long time period (2013–2021). We therefore endorse collection of results from larger registries (e.g.: Institut für Qualität und Patientensicherheit, URL https://www.bqs.de/ or Deutsche Gesellschaft für Interventionelle Radiologie und minimalinvasive Therapie, URL https://www.degir.de/), separately analysing emergency (± MT) and elective CAS cases as these procedures are difficult to compare; however, we believe that we provide detailed first data for novel DRLs in various CAS situations that can be used as a stepstone for large multicenter studies as comparable data are scarce. Furthermore, at least the number of elective CAS patients in our study (*n* = 75) may indeed be sufficient, as Miller et al. recommended at least 30 studies of the same procedure for definition of local DRLs [25]. Third, the following data were not collected: Kerma area product, type of aortic arch, severity of ECS according to NASCET criteria, PTA maneuver count in the 19 combined CAS + MT procedures (due to the relatively low sample size and consequently missing statistical power of multivariate analysis), use of a distal protection device, influence of the operator. Furthermore, complications (e.g., iatrogenic dissections, dislocation of the stent applicator, etc.) and cases with additional 3D rotational angiography were excluded but might have substantially influenced dosimetrics. Finally, modern soft-/hardware techniques (e.g., ClarityIQ, Philips or OPTIQ, Siemens Healthineers) had not yet been installed within the study period. A dedicated technical upgrade implies a great potential for further reduction of radiation exposure, e.g., by automatic adjustment of tube parameters for dose efficiency optimization with variable detector entrance dose, or tuning of the X‑ray spectrum for the purpose of material-specific imaging [30].

In conclusion, we suggest novel local DRLs for fluoroscopically guided endovascular CAS due to ECS, considering elective and emergency indications with or without intracranial thrombectomy in case of acute tandem occlusion. Our results may be valuable for a reasonable extension of the existing DRL catalogue. DAP and fluoroscopy times were highest and lowest for combined CAS + MT and semielective/elective CAS-only procedures, respectively. Dosimetry values increased significantly in patients undergoing more than one additional PTA maneuver. The etiology of ECS, number of applied stents and MT maneuvers had no significant impact on dosimetrics. A dedicated low-dose protocol yielded a 33% reduction of radiation exposure. Prospective, ideally multi-centric studies with larger data collections are warranted in the future, particularly considering different angiography systems of various manufacturers and modern dose reduction platforms.
